# Ultra-weak photon emission as a dynamic tool for monitoring oxidative stress metabolism

**DOI:** 10.1038/s41598-017-01229-x

**Published:** 2017-04-27

**Authors:** Rosilene Cristina Rossetto Burgos, Johannes Cornelius Schoeman, Lennart Jan van Winden, Kateřina Červinková, Rawi Ramautar, Eduard P. A. Van Wijk, Michal Cifra, Ruud Berger, Thomas Hankemeier, Jan van der Greef

**Affiliations:** 1 Division of Analytical Biosciences, Leiden Academic Centre for Drug Research, Leiden University, P.O. Box 9502, 2300 RA Leiden, The Netherlands; 2 Sino-Dutch Centre for Preventive and Personalised Medicine/Centre for Photonics of Living Systems, Leiden University, P.O. Box 9502, 2300 RA Leiden, The Netherlands; 3 Institute of Photonics and Electronics, The Czech Academy of Sciences, Chaberská 57, 182 00 Prague, Czech Republic; 4 Faculty of Electrical Engineering, Czech Technical University in Prague, Technická 2, 166 27 Prague, Czech Republic

## Abstract

In recent years, excessive oxidative metabolism has been reported as a critical determinant of pathogenicity in many diseases. The advent of a simple tool that can provide a physiological readout of oxidative stress would be a major step towards monitoring this dynamic process in biological systems, while also improving our understanding of this process. Ultra-weak photon emission (UPE) has been proposed as a potential tool for measuring oxidative processes due to the association between UPE and reactive oxygen species. Here, we used HL-60 cells as an *in vitro* model to test the potential of using UPE as readout for dynamically monitoring oxidative stress after inducing respiratory burst. In addition, to probe for possible changes in oxidative metabolism, we performed targeted metabolomics on cell extracts and culture medium. Lastly, we tested the effects of treating cells with the NADPH oxidase inhibitor diphenyleneiodonium chloride (DPI). Our results show that UPE can be used as readout for measuring oxidative stress metabolism and related processes.

## Introduction

Respiratory burst is one of the first defence mechanisms used by specialised cells such as neutrophils in response to invading pathogens^1–3^. This process uses the rapid consumption of molecular oxygen (O_2_) to produce high levels of intracellular reactive oxygen species (ROS) for killing invading pathogens^1–3^. Under homeostatic conditions, ROS are produced by mitochondria as a product of cellular metabolism^4^; however, during respiratory burst NADPH oxidase plays a central role in ROS production for cellular defence^5, 6^. The primary function of NADPH oxidase is the production of superoxide radicals (O_2_^·^^−^)^7, 8^, which serve as the initial substrate in the generation of a diverse variety of ROS species, including hydrogen peroxide (H_2_O_2_) and hydroxyl radicals (OH·).

Physiologically, ROS have a hormetic effect ‒ at relatively low concentrations, ROS have beneficial properties, which include maintaining cellular redox biology and facilitating signalling^9^, whereas at high concentrations, ROS cause oxidative stress that can damage nucleic acids, proteins, and lipids. Studies have shown that oxidative stress contributes to the pathogenesis of many diseases and conditions, including chronic inflammation^10^, various types of cancers^11^, Alzheimer’s disease^12^, and cardiovascular disease^13^. Moreover, the aforementioned studies revealed a clear association between ROS production and NADPH oxidase, thereby providing insight into the underlying ROS-based physiological processes. In recent years, several NADPH inhibitors have been investigated as candidate therapies for ROS-related pathology^14, 15^, and measuring these oxidation reactions and related biomolecules can provide a readout of cellular oxidative stress^16^. ROS production has been analysed using various techniques, including photometry, luminometry, flow cytometry, and precipitation reactions^17^. However, all of these techniques provide a measure at only a single time point or require labels; moreover, these techniques are cell-dependent, laborious, and not necessarily feasible for diagnostic purposes. In contrast, ultra-weak photon emission (UPE) is a promising new tool that could be used to monitor oxidative processes. Indeed, the feasibility of using UPE as a tool for monitoring health and disease has been examined in several studies^18–20^.

Ultra-weak photons are emitted spontaneously by many biological systems^21, 22^. UPE is characterised as non-thermal radiation in the near-ultraviolet to visible region (100–800 nm) of the electromagnetic spectrum, possibly reaching the near-infrared region (801–1300 nm). UPE is generated by the transition of electrons from an excited state to the ground state; excited electron states (e.g. triplet carbonyls, singlet oxygen, etc.) are produced by the oxidation of biomolecules by ROS^21, 23^. Thus, UPE is a potential new tool for monitoring dynamic biological processes that involve ROS, including ROS-related diseases^24, 25^, as well as processes related to oxidative stress metabolism. A clear advantage of UPE is that it provides spatiotemporal information; in addition, UPE is non-damaging, non-invasive, label-free, and relatively cost-effective.

Because UPE can reflect complex molecular processes, it can be combined with other technologies such as metabolomics, thereby providing valuable insight into the biochemical processes probed using UPE^26^. Recently, we used metabolomics to identify several metabolites correlated with UPE^27^. Moreover, metabolomics is a powerful approach that can be applied to numerous biological studies due to the ability to detect many hundreds of metabolites in a single biological sample, thereby providing a ‘phenotypic’ readout of other ‘omics’. Previous reports suggest that lipid peroxidation of linoleic acid in cell membranes can strongly affect UPE emission^18, 28^. In this respect, the products of lipid and protein oxidation are closely related to UPE. For example, compounds related to the arachidonic acid pathway (e.g. isoprostanes, prostaglandins, and lysosphingolipids) are key signalling compounds in biological systems and are often related to oxidative stress and/or inflammatory processes^29, 30^. In addition, previous studies demonstrated the relationship between these metabolites and low-level chemiluminescence and electronically excited species^31–34^.

Here, we evaluated the feasibility of using UPE as a dynamic tool for monitoring oxidative stress metabolism and related processes. A metabolomics approach was also used to gain insight into the biochemical processes probed using UPE. We used differentiated neutrophil-like HL-60 cells as an *in vitro* model. Respiratory burst was induced by treating the cells with phorbol 12-myristate 13-acetate (PMA)^35, 36^; in response to PMA, these cells produce large quantities of ROS, which were monitored in real-time using UPE. A targeted metabolomics approach was then used to analyse metabolites related to oxidative stress and inflammation (e.g. prostaglandins and isoprostanes) in HL-60 cell extracts and culture medium. Moreover, we measured the effects of treating cells with the NADPH oxidase inhibitor diphenyleneiodonium chloride (DPI).

## Results

### Ultra-weak photon emission (UPE)

**Figure 1:**
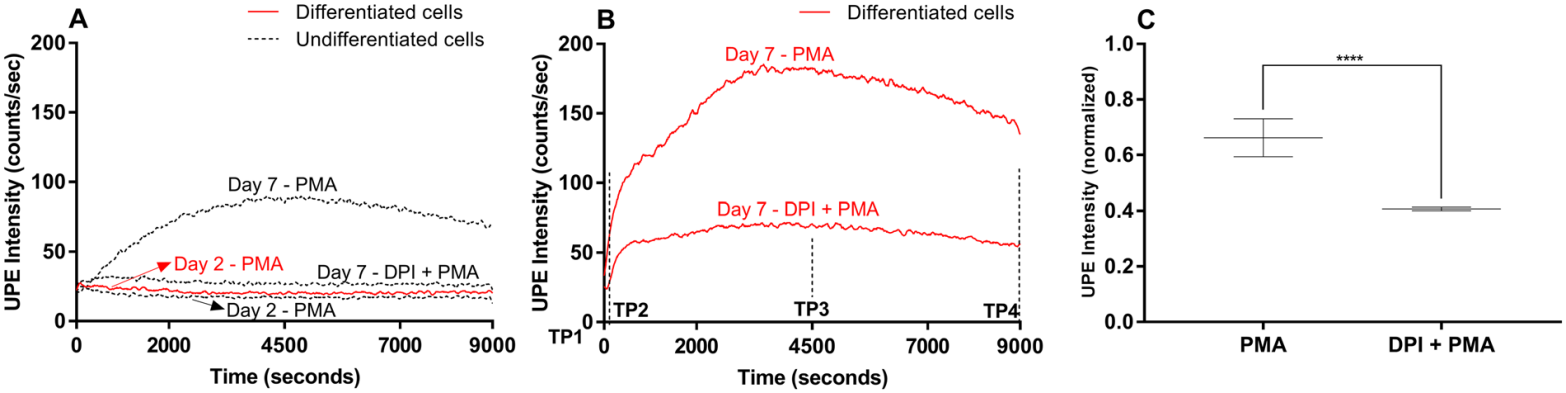
Dynamic UPE measurement of HL-60 cells treated with ATRA for 2 or 7 days to differentiate the cells into neutrophil-like cells. The lines (red - differentiated cells and black - undifferentiated cells) represent the moving UPE average of 100 points. **(A)** Control measurements. **(B)** The UPE profile of differentiated cells (day 7) was recorded after stimulation with PMA in the absence or presence of DPI. **(C)** Summary of peak UPE intensity measured in HL-60 cell treated for 7 days with ATRA after stimulation with PMA in the absence or presence of DPI. The data are represented as the mean ± SD (n = 5) of the normalized maximum peak intensity. Student’s paired *t*-test with ****p < 0.0001.

HL-60 cells were differentiated into neutrophil-like cells by incubation in all-*trans* retinoic acid (ATRA) for up to 7 days. Next, we induced respiratory burst by treating the cells with PMA. After PMA induction we measured the dynamic UPE profile for 9000 seconds (Fig. 1). Figure 1A shows control experiments recorded under the same conditions and Fig. 1B presents the UPE profile of differentiated cells on Day 7 induced by PMA in the presence or absence of DPI. Figure 1C illustrates the paired t-test performed (*n* = *5*).

Cells treated with ATRA for only 2 days had no response to PMA stimulation. In contrast, cells treated with ATRA for 7 days had a robust increase in UPE in response to PMA due to a high amount of ROS generated. This profile is in agreement with our previous work^27^. Treating the differentiated cells with the NADPH oxidase inhibitor DPI significantly reduced (*p* < 0.0001) the UPE response, substantiating the biochemical link between ROS and UPE. Furthermore, this inhibition was also observed in undifferentiated cells (see Fig. 1A **)** due to a small percentage of cells spontaneously differentiating into neutrophils-like cells^27^.

### Metabolic profiling of isoprostanes, prostaglandins and lysosphingolipids

Next, to measure the biochemical changes related to oxidative metabolism, we performed targeted metabolomics of compounds related to oxidative stress and inflammation, including prostaglandins, isoprostanes, nitro-fatty acids, and lysosphingolipids. We used our previously reported approach^27^ (see also Figure S1), and DPI treatment was included in order to determine which metabolic pathways may be involved in this process.

Based on the dynamic UPE profiles (see Fig. 1B and Figure S1), cell lysates (to measure intracellular metabolites) and culture medium samples (to measure extracellular metabolites) were obtained at four time points relative to PMA stimulation and analysed using targeted metabolomics. TP1 corresponds to the basal condition (i.e. prior to the addition of PMA), and TP2, TP3 and TP4 correspond to 60, 4500 and 9000 seconds after PMA stimulation, respectively. To measure the metabolic profiles indicative of oxidative stress and inflammation, we used a custom-built liquid chromatography‒mass spectrometry platform^37^. A total of 10 and 11 metabolites were detected in the cell lysates and culture medium samples, respectively; 7 metabolites were detected in both samples (Table S1).

#### Intracellular metabolites

**Figure 2:**
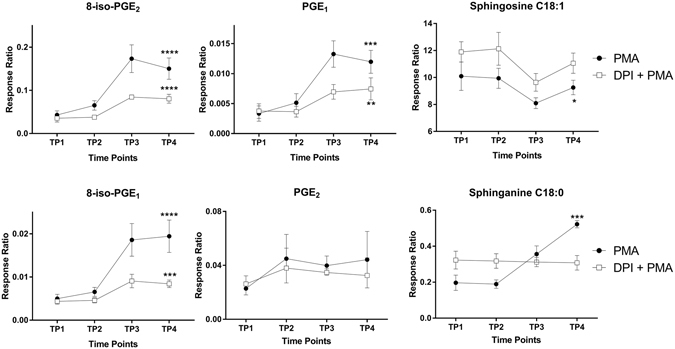
HL-60 cells were treated with ATRA for 7 days, after which respiratory burst was induced with PMA in the presence or absence of DPI. Cell lysates were collected at the indicated time points, and intracellular metabolites were measured. We performed intragroup ANOVA analysis over the four time points to identify significant changes in metabolite levels. Data are plotted as the mean ± SD (n = 3). ANOVA with *p < 0.05, **p < 0.01, ***p < 0.001, and ****p < 0.0001.

Among the 10 intracellular metabolites measured in the cell lysates, only 8-iso-PGE_2_, 8-iso-PGE_1_, PGE_1_ and sphinganine C18:0 increased significantly in response to PMA stimulation, and sphingosine C18:1 significantly decreased in response to PMA stimulation shown in Fig. 2 and Table S2. PGE_2_ did display an increasing trend over the four time points. We also found a significant correlation between the recorded UPE data and the metabolites measured (see Table S2).

Pre-treating the cells with DPI significantly decreased the PMA-induced responses of 8-iso-PGE_2_, 8-iso-PGE_1_ and PGE_1_. In addition, the PMA-induced response of sphingosine increased, whereas sphinganine C18:0 had no response (see Fig. 2).

#### Extracellular metabolites

**Figure 3:**
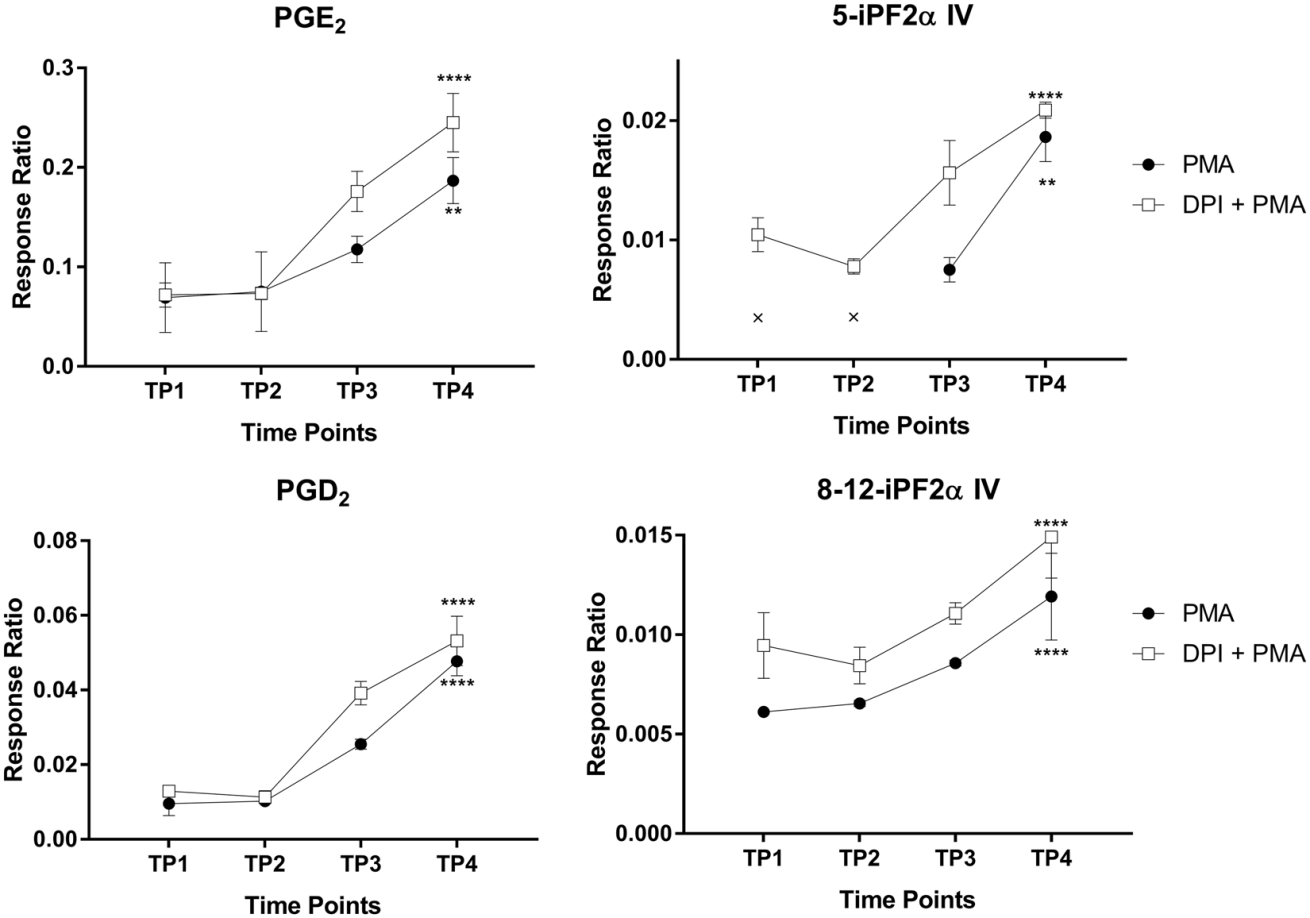
HL-60 cells were treated with ATRA for 7 days, after which respiratory burst was induced with PMA in the presence or absence of DPI. Culture medium was collected at the indicated time points, and extracellular metabolites were measured. We performed intragroup ANOVA analysis over the four time points to identify significant changes in metabolite levels. Data are plotted as the mean ± SD (n = 3/group). ANOVA with **p < 0.01 and ****p < 0.0001. The data points indicated with an “x” in the top-right panel were below the metabolite’s limit of detection.

Next, we examined the extracellular metabolites measured from the culture medium during PMA-induced respiratory burst. Among the 11 compounds detected (see Table S1 **)**, only PGE_2_, PGD_2_, (±)5-iPF2α-IV, and 8-12-iPF2α-IV increased significantly during respiratory burst (Fig. 3 and Table S3). Interestingly, treating the cells with DPI also increased the extracellular levels of all four compounds. Spearman’s correlation showed a high correlation coefficient (r > 0.6) to the extracellular metabolite levels and the measured UPE intensity. However, the *p-values* were not significant (*p* > 0.05).

## Discussion

**Figure 4:**
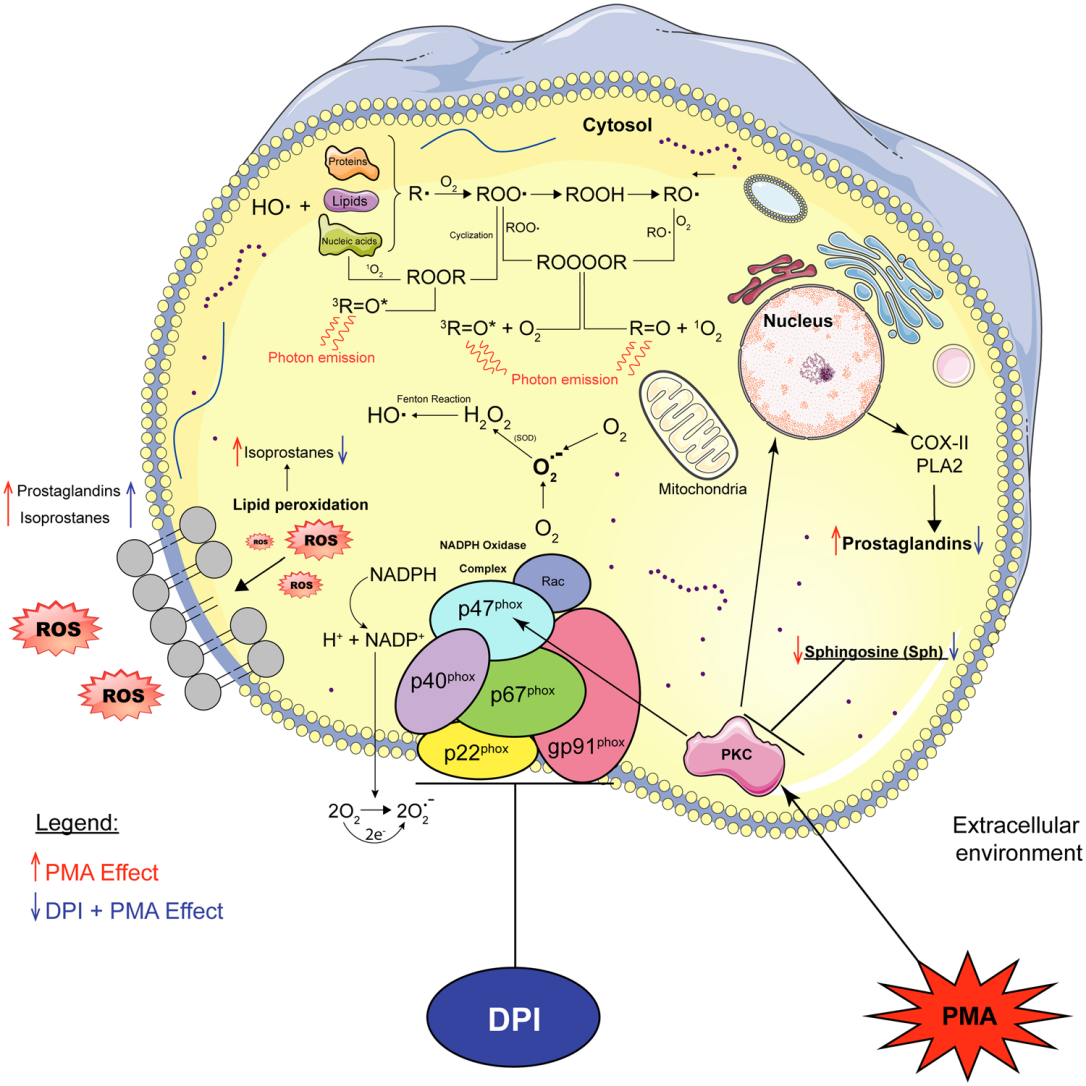
Schematic overview of the biological events involved in NADPH oxidase during PMA-induced respiratory burst in differentiated HL-60 cells. PMA activates protein kinase C (PKC), which signals to the nucleus, activating cyclooxygenase (COX) and phospholipase A_2_ (PLA2) pathways, leading to the production of prostaglandins. O_2_^.−^is first produced by NADPH oxidase as a primary ROS and is subsequently dismutated to H_2_O_2_ by superoxide dismutase (SOD). Thus, H_2_O_2_ serves as a substrate for generating hydroxyl radicals (OH^•^) via the Fenton reaction. Hydroxyl radicals (OH^•^) are potent oxidants that can produce the initial radical (R•) form of a wide range of biomolecules, including lipids, proteins, and nucleic acids. Via this mechanism, molecular oxygen is added to produce a peroxyl radical (ROO^•^), followed by cyclisation to produce dioxetane (ROOR) and decomposition to produce triplet excited carbonyl^23^. Alternatively, two ROO^•^ moieties can recombine to form tetroxide (ROOOOR), which can decompose to form triplet excited carbonyl or singlet oxygen via the Russel reaction^23^. These electron-excited species emit photons, giving rise to UPE^23^. Eventually, intracellular ROS can react with biomolecules in the cell membrane (i.e. lipid peroxidation), giving rise to isoprostanes. Treating differentiated HL-60 cells with DPI, which binds to the NADPH oxidase complex, partially inhibits ROS production, decreases UPE emission, and decreases the levels of prostaglandins and isoprostanes. This figure was drawn by the first author R.C.R. Burgos using the software Adobe Illustrator and the image bank of Servier Medical Art. Servier Medical Art by Servier is licensed under a Creative Commons Attribution 3.0 Unported License. https://creativecommons.org/licenses/by/3.0/.

Treating HL-60 cells with ATRA causes the cells to differentiate into neutrophil-like cells^35^. Neutrophils are specialised cells capable of producing high amounts of ROS. In addition, the differentiation of HL-60 cells into neutrophil-like cells is associated with morphological changes, as well as metabolic changes that occur at the cell surface and in the nucleus^35, 38, 39^. We used PMA to induce respiratory burst, thereby activating protein kinase C (PKC). PKC phosphorylates the cytosolic NADPH oxidase subunit p47^PHOX^, contributing to assembly of the NADPH oxidase complex^40–42^. The function of NADPH oxidase is to extract electrons from NADPH, transferring the electrons to oxygen, thereby forming O_2_^·^^−^ in the cytosol and extracellular space; this ultimately leads to the generation of other ROS species^5–7^. Here, we found that UPE can be used to establish the link between respiratory burst and increased levels of ROS in response to PMA stimulation (Fig. 4).

The NADPH oxidase inhibitor DPI binds specifically to the flavoprotein subunit (a polypeptide in the plasma membrane - cytochrome b558), thereby blocking the flow of electrons in the NADPH oxidase complex^5, 6, 43–45^. DPI has also been used to inhibit mitochondrial ROS production^46^. In our study, DPI significantly decreased the UPE signal in PMA-stimulated differentiated HL-60 cells. Interestingly, DPI also acts by suppressing O_2_^·^^−^ production, H_2_O_2_ production, and mitochondrial processes such as NADH-ubiquinone oxidoreductase (complex I)^43, 44, 46^ (see Fig. 4). In our study, we used DPI at its reported IC_50_ value(0.9 µM)^44^. At this concentration, DPI caused a 50–60% decrease in the UPE signal, suggesting residual electron transport flow, likely due to residual NADPH oxidase activity and the relatively low levels of photon emissions from mitochondrial activity.

With respect to intracellular metabolites, isoprostanes such as 8-iso-PGE_2_ and 8-iso-PGE_1_ are non-enzymatic products produced by the auto-oxidation of arachidonic acid by free radicals; thus, these metabolites are useful markers of oxidative stress^30^. In biological processes involving ROS, polyunsaturated fatty acids (PUFAs) ‒ particularly arachidonic acid ‒ are susceptible to oxidation by free radicals. PUFAs also serve as a precursor for cyclooxygenase-mediated oxidation, producing thromboxanes, prostacyclins, and prostaglandins^30, 47, 48^.

Although the production of the isoprostane 8-iso-PGE_1_ is poorly understood, one study reported that E_1_-isoprostane is produced in plants by oxidation, with α-linoleic acid as the substrate^49^. Similarly, little is known regarding the production and biological role of 8-iso-PGE_2_. In a previous *in vivo* study with rats, 8-iso-PGE_2_ was identified as a non-enzymatic product of free radical‒catalysed lipid peroxidation, ultimately exerting potent biological activity (in this case, renal vasoconstriction)^50^. Another biological role of 8-iso-PGE_2_ is the ability to inhibit platelet aggregation^51^. Thus, 8-iso-PGE_2_ appears to act as a signalling molecule with a wide range of biological functions.

The prostaglandins PGE_1_ and PGE_2_ have been well characterised and mediate a variety of biological processes, including vasodilation^52^, platelet aggregation^53^, and adaptive crosstalk activation^54^. Here, we found that the intracellular levels of both 8-iso-PGE_2_ and 8-iso-PGE_1_ are increased during respiratory burst in differentiated HL-60 cells; therefore, these metabolites are presumably products of ROS oxidation mediated by NADPH oxidase and mitochondrial electron transport. Moreover, pre-treating cells with DPI significantly reduced the intracellular levels of isoprostanes and prostaglandins (see Fig. 2). Taken together, our results show that DPI treatment significantly decreases the intracellular levels of 8-iso-PGE_1_, 8-iso-PGE_2_, and PGE_1_, thereby showing a clear correlation with UPE intensity.

Sphingosine and sphinganine are lysosphingolipids that serve as precursors for ceramide and/or sphingosine-1-phosphate, both of which are involved in various signalling pathways, including cell proliferation, differentiation, and apoptosis^55^. In addition, both sphingosine and sphinganine can inhibit PKC activity^56, 57^ (see Fig. 4). PKC phosphorylates p47^PHOX^, a key component of NADPH oxidase (see Fig. 4), and therefore contributes to the activation of respiratory burst by inducing flavoenzyme (cytochrome b558)^44^. Our finding of increased levels of sphinganine, however, suggests that PKC activity has an inhibitory role, acting as a negative regulator of respiratory burst. During respiratory burst, sphingosine C18:1 decreased significantly (see Fig. 2). Previous studies found that sphingosine is converted to *N*,*N*-dimethyl sphingosine (DMS), which has an even stronger inhibitory effect on PKC^58^. This may explain the decrease in sphingosine C18:1; however, DMS was not included in our analysis.

E-series prostaglandins play a role in cell signalling by activating E2 and E4 prostanoid receptors located on the neutrophils^47^. In a negative feedback mechanism, receptor E2 and E4 activation increases intracellular levels of cAMP inhibiting neutrophil extracellular traps^59^ and the respiratory burst^60^. This may explain the increased extracellular levels of E-series prostaglandins, including PGE_2_ as a protective mechanism.

PGD_2_ has both pro-inflammatory and anti-inflammatory properties and is considered one of the major mediators of the mast cell allergic response and can serve as a potent eosinophil chemoattractant^61, 62^. During respiratory burst, PGD_2_ is rapidly metabolised through enzymatic and non-enzymatic pathways to 11-epi-PGF2α, dihydro-15-keto-PGD_2_, and PGJ_2_ in order to attract eosinophils to inflammatory sites^61^. However, PGJ_2_ was not included among the metabolites analysed in the current study. Thus, PGD_2_ may be produced during respiratory burst in order to attract other immune cells (e.g. eosinophils), thereby further stimulating the immune response at the site of inflammation. We hypothesise that ROS generated by NADPH oxidase during respiratory burst induces lipid peroxidation in the cell membrane. Thus, prostaglandins and isoprostanes are secreted into the extracellular space during membrane repair (see Fig. 4). Although our analysis revealed significant PMA-induced changes in PGE_2_, PGD_2_, (±)5-iPF2α-IV, and 8-12-iPF2α-IV in the medium, DPI had the same effect, with slightly increased levels.

Taken together, these results suggest that UPE is correlated only with intracellular signalling metabolic intermediates. These findings strongly support the notion that UPE is linked to intracellular metabolism. Another explanation for the results of our analysis of extracellular metabolites is that the time course of respiratory burst (measured up to 9000 seconds) was too short for studying the flow of metabolites from the cytoplasm to the extracellular medium.

In summary, we report a strong correlation between ultra-weak photon emission intensity, NADPH oxidase activity and intracellular metabolism. The intracellular levels of the isoprostanes 8-iso-PGE_1_ and 8-iso-PGE_2_ and the prostaglandin PGE_1_ significantly increased during PMA-induced respiratory burst, and DPI inhibited 50–60% of the PMA-induced UPE signal. These results indicate that UPE can be used as a dynamic readout tool in combination with metabolomics to monitor oxidative metabolism in ROS-related physiological processes. Follow-up studies should focus on identifying the specific radical species using spin-trapping electron paramagnetic resonance, which may also help identify the molecules that undergo oxidative damage. Optical spectral analysis of the UPE signal may also help identify the specific photon-emitting molecules.

## Methods

### Cell culture and experimental design

All experiments with human cell lines were performed in accordance with approved guidelines, and all experimental protocols were approved in accordance with the regulations established by the Institute of Photonics and Electronics, Czech Academy of Sciences. The acute promyelocytic leukaemia cell line HL-60 (catalogue number CCL-240; lot number 62690063; ATCC, Manassas, VA) were cultured in Iscove’s Modified Dulbecco’s Medium (IMDM) without phenol red (Gibco-Life Technologies, Grand Island, NY) supplemented with 10% (v/v) fetal calf serum and 1% (v/v) penicillin/streptomycin (Sigma-Aldrich, St. Louis, MO) in an incubator at 37 °C in 5% CO_2_. The cells were seeded at 2 × 10^5^ cells/ml and maintained in the exponential growth phase in accordance with the instructions provided by ATCC. Cell number and viability were measured using the trypan blue exclusion method with an automated cell counter (Bio-Rad Laboratories, Hercules, CA). Cell viability was >85%. UPE and metabolomics measurements were performed at cell passage number 28. We have used the experimental design as described previously^27^, with minor modifications. In brief, when the cells were split and adjusted for cell density, 1 µM all-*trans* retinoic acid (ATRA; 98% grade, catalogue number R250, Sigma-Aldrich) was added to the cells in order to induce differentiation via the granulocytic pathway; control cells received the same volume of vehicle. The cells were then incubated for up to 7 days, and UPE and metabolomics experiments were performed on days 2 and 7. Prior to any UPE measurement and/or sample collection for metabolomics, the culture medium was replaced with fresh IMDM (without supplementation), and the cells were counted. Where indicated, cells were stimulated with phorbol 12-myristate 13-acetate (PMA; 98% grade, Sigma-Aldrich) in the presence or absence of diphenyleneiodonium chloride (DPI; Cayman Chemicals, Ann Arbor, MI). A small aliquot of the cell suspension was used for UPE measurements. Based on the UPE profile, four time points were used for the metabolomics study; aliquots containing of 12 × 10^6^ cells were used for each time point. TP1 samples were collected prior to PMA induction; TP2, TP3, and TP4 samples were collected 60, 4500, and 9000 seconds, respectively, after PMA induction (see Figure S1).

### Ultra-weak photon emission (UPE)

UPE was measured using a module H7360-01 photomultiplier tube (PMT; Hamamatsu Photonics, Hamamatsu, Japan), which is sensitive to wavelengths of 300–650 nm and has a dark count of approximately 13 counts per second. UPE was measured from HL-60 cells suspended in IMDM without any supplementation. The cell suspension (3 ml containing 1.5 × 10^6^ cells/ml) was transferred to a small Petri dish, which was then placed in the PMT dark chamber at 37 °C. The Petri dish was positioned as close as possible to the PMT detector using a sample holder. A background measurement was taken before each sample measurement. To stimulate the respiratory burst in cells, PMA was applied at a concentration of 54 nM, and the UPE profile was recorded for 9000 seconds. DPI was applied at a concentration of 0.9 µM prior to PMA (54 nM) stimulation in independent cell suspensions after which the UPE profile was recorded for 9000 seconds. There was a brief delay of approximately 30 seconds between PMA application and the start of the UPE measurement due to placing the samples in the PMT dark chamber. Control cells (i.e. cells that were not cultured in ATRA) were measured using the same conditions.

### Collection, quenching, and extraction of cell pellets and culture medium for metabolomics

#### Sample collection and quenching

Aliquots of suspension cells were centrifuged for 4 minutes at 0.2 rcf at room temperature. The supernatant (containing the culture medium) was collected and stored at −80 °C. The pellets (containing the cells) were quenched in 0.9% (w/v) sodium chloride solution (Sigma-Aldrich) at 0–2 °C. The cell aliquots were then centrifuged for 4 minutes at 0.2 rcf at room temperature. The supernatant was discarded, and the cell pellets were stored at −80 °C.

#### Extraction

The medium and cell pellets were extracted using a liquid-liquid extraction protocol as described below.

*Cell pellets*–First, each cell pellet was suspended in 500 µl citric acid (Merck, Darmstadt, Germany), 5 µl antioxidant containing 0.4 mg/ml butylated hydroxytoluene (BHT; Sigma-Aldrich) and 0.4 mg/ml ethylenediaminetetraacetic acid (EDTA; Sigma-Aldrich), 10 µl internal standard mix (ISTD) comprised of deuterated compounds (Cayman Chemicals), and 1 ml n-butanol (Boom B.V., Meppel, the Netherlands):ethyl acetate (Biosolve B.V., Valkenswaard, the Netherlands). The cell suspension was shaken in a bullet blender (Next Advance, Averill Park, NY) to mix the organic phase and lyse the cells. The suspension was then centrifuged for 10 minutes at 16.1 rcf at 4 °C, and 900 µl of the organic phase was collected from each sample. The remaining mixture was subjected to a second extraction round with 400 µl n-butanol (saturated in Milli-Q water from EMD Millipore, Billerica, MA) and 400 µl of ethyl acetate. The mixture was then shaken and centrifuged, and 800 µl of the organic phase was collected and added to the first-round organic phase sample. Finally, the organic phase was dried in a CentriVap centrifugal concentrator. The dried samples were resuspended in 30 µl injection solution consisting of 70% MeOH (Biosolve B.V.) in Milli-Q water and transferred to a glass vial suitable for use in the LC system.

*Medium samples*–Medium samples (800 µl total) were divided into two 400-µl aliquots to increase the concentration of extracellular metabolites. First, we added 400 µl citric acid, 5 µl antioxidant (0.4 mg/ml BHT and 0.4 mg/ml EDTA), 6 µl ISTD mix, and 1 ml butanol:ethyl acetate to each aliquot. The samples were then shaken using a bullet blender, centrifuged for 10 minutes at 16.1 rcf at 4 °C, and 950 µl of the organic phase was collected. The remaining mixture was subjected to a second extraction round with 500 µl butanol saturated in Milli-Q water and 500 µl of ethyl acetate. The mixture was then shaken and centrifuged for 10 minutes at 16.1 rcf at 4 °C; 950 µl of the organic phase was collected and added to the first-round organic phase sample. The organic phase was dried using a CentriVap centrifugal concentrator. The dried samples were resuspended in 30 µl injection solution (70% MeOH in Milli-Q water) and transferred to a glass vial suitable for use in the LC system.

#### Liquid chromatography–mass spectrometry (LC-MS) analysis

For metabolomics, a model LCMS-8050 liquid chromatograph-mass spectrometer (Shimadzu, Tokyo, Japan) and an ACQUITY BEH C18 column (50 mm × 2.1 mm, 1.7 μm; Waters, Milford, MA) maintained at 40 °C was used for reverse-phase LC separation. Electrospray ionisation was used as an ionisation source. The mobile phase A consisted of H_2_O containing 0.1% acetic acid. The mobile phase B contained 75% acetonitrile, 25% MeOH, and 0.1% acetic acid (Sigma-Aldrich). The mobile phase C contained 100% isopropanol. The temperature of the column was set at 40 °C, and the temperature of the autosampler was set at 5 °C. The injection volume and flow rate were 10 µl and 0.7 ml/min, respectively. After each measurement, peak detection and integration of the raw data were processed and analysed using the Shimadzu Lab Solutions software program, version 5.65. Pooled cell pellet extracts and pooled medium samples were prepared and measured after every ten samples in order to verify reliability of the measurements. Any metabolites detected in the pooled samples that exceeded 30% of the relative standard deviation due to technical and/or analytical variations were excluded from analysis.

### Statistical analysis

UPE data were analysed using GraphPad Prism (GraphPad Software, Inc., La Jolla, CA). The two-tailed paired Student’s *t*-test was used to compare individual groups. The metabolic results (i.e. response ratio) were uploaded to the MetaboAnalyst website (http://www.metaboanalyst.ca)^63, 64^ and tested for statistical significance. To obtain data with a normal distribution, the metabolomics data were log-transformed and auto-scaled. We analysed the following parameters: (*i*) the variance between the four time points (i.e. TP1, TP2, TP3, and TP4) relative to PMA stimulation was analysed using a two-way ANOVA (two-tailed); (*ii*)) the variance between the four time points in cells treated with DPI and PMA were analysed using a two-way ANOVA (two-tailed); and (*iii*)) Spearman’s correlation coefficient (two-tailed) was used to analyse the relationship between the UPE data and metabolic profile. Spearman’s correlations were done independently for the PMA and DPI + PMA groups, correlating their averaged metabolite levels to their respective averaged UPE profiles over the four TPs measured. Differences with a *p*-value < 0.05 were considered significant.

## Electronic supplementary material

Ultra-weak photon emission as a dynamic tool for monitoring oxidative stress metabolism
