# Supplementary material for: Ultra-weak photon emission as a dynamic tool for monitoring oxidative stress metabolism
Source: Sci Rep. 2017 Apr 27;7:1229. doi: 10.1038/s41598-017-01229-x (PMC5430737; doi:10.1038/s41598-017-01229-x)
Supplement: Supplementary file 1 — Ultra-weak photon emission as a dynamic tool for monitoring oxidative stress metabolism [file 41598_2017_1229_MOESM1_ESM.pdf]

Supplementary Information

Ultra-weak photon emission as a dynamic tool for monitoring oxidative stress metabolism.

Rosilene Cristina Rossetto Burgos, Johannes Cornelius Schoeman, Lennart Jan van Winden, Kateřina Červinková, Rawi Ramautar, Eduard P.A. Van Wijk, Michal Cifra, Ruud Berger, Thomas Hankemeier, Jan van der Greef.

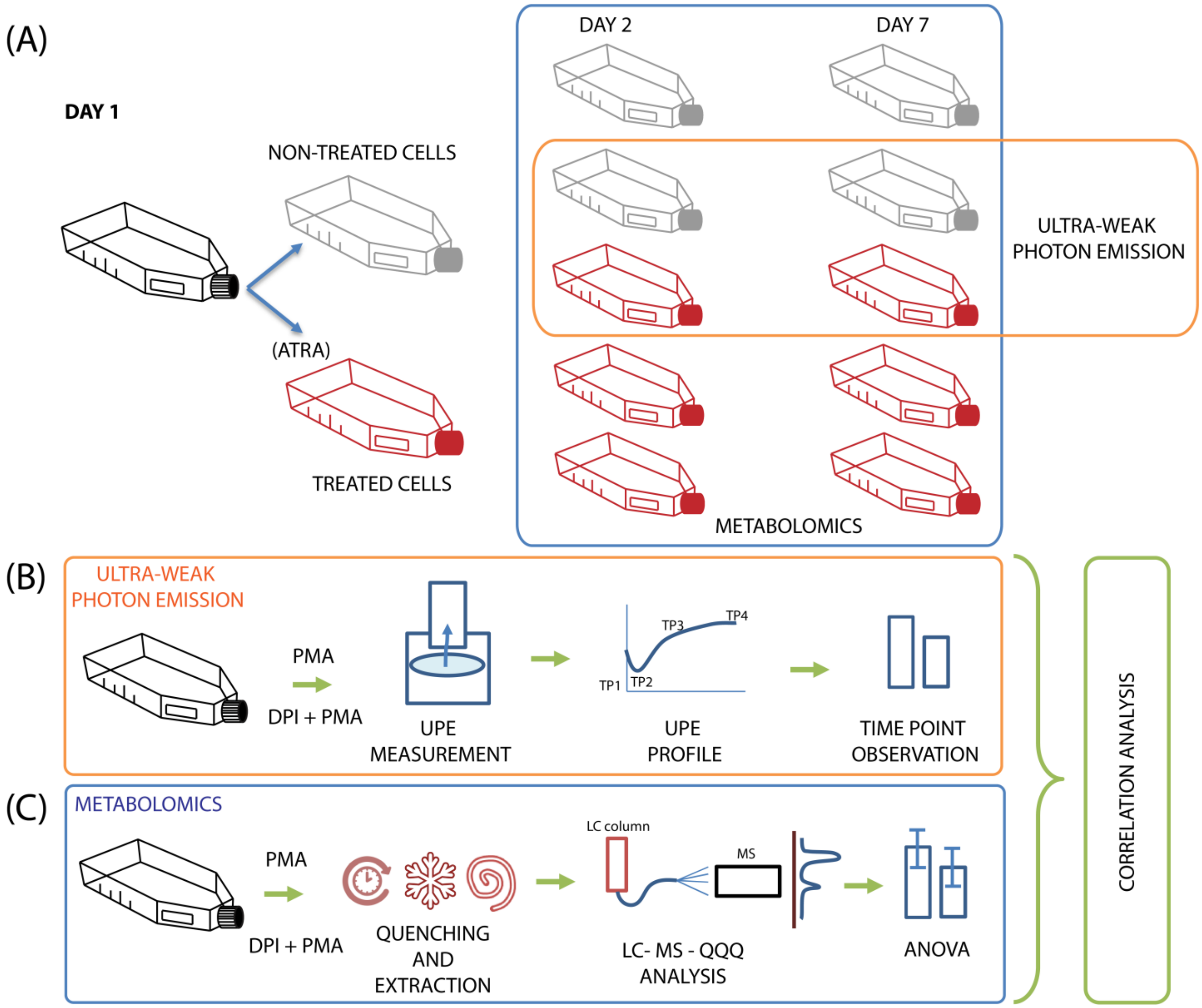

Supplementary Figure S1: Experimental design. (A) Cultured HL-60 cells were split into 10 flasks; six flasks were treated with ATRA (red), and four flasks received only vehicle (grey). (B) Ultra-weak photon emission (UPE) was measured on days 2 and 7. PMA or DPI+PMA was applied to each flask to induce respiratory burst, and UPE was recorded for 9000 seconds. (C) Samples were collected for metabolomics analyses at the specified time points (TP1, TP2, TP3, and TP4) indicated in (B). TP1 samples were collected prior to the addition of PMA/ DPI. After sample collection, liquid-liquid extraction was used to obtain cell extracts and medium extracts. The metabolic profile was recorded using LC-MS-QQQ. The metabolic data were analysed using an ANOVA, and Spearman's correlation coefficient was calculated in order to analyse the relationship between the UPE data and metabolic profiles.

Supplementary Table S1: List of metabolites detected in the intracellular (cell lysates) and extracellular (culture medium) samples.

METABOLITE	INTRACELLULAR ANALYSIS	EXTRACELLULAR ANALYSIS
8-iso-PGE_2	Detected	Not detected
8-iso-PGE_1	Detected	Not detected
PGE_2	Detected	Detected
PGE_1	Detected	Detected
PGD_2	Not detected	Detected
PGF_{2a}	Detected	Detected
(±)5-iPF2a-IV	Not detected	Detected
8-iso-PGA_1	Detected	Detected
PGA_2	Detected	Detected
8-12-iPF2a-IV	Detected	Detected
Sph C18_1	Detected	Not detected
Spha C18_0	Detected	Detected
NO_2-LA	Not detected	Detected

PGE, prostaglandin E; PGD, prostaglandin D; PGF, prostaglandin F; iPF, isoprostane F; PGA, prostaglandin A; Sph, sphingosine C18:1; Spha, sphinganine C18:0; NO2-LA, nitro-linoleic acid.

Supplementary Table S2: Intracellular metabolites with significant changes during PMA and DPI+PMA treatment (n=3/ TP) collected on Day 7.

	ANOVA				SPEARMAN'S CORRELATION	
Sample Treatment	*Compound*	*p-value*	*FDR* [a]	*Fisher's LSD* [b]	*Corr. Coefficient*	*p-value*
**PMA**	**Sphinganine C18:0**	3.93E-05	0.00017	TP3 vs TP1; TP4 vs TP1; TP3 vs TP2; TP4 vs TP2; TP4 vs TP3	0.60	0.40
	**8-iso-PGE1**	4.23E-05	0.00017	TP3 vs TP1; TP4 vs TP1; TP3 vs TP2; TP4 vs TP2	0.80	0.20
	**8-iso-PGE2**	4.51E-05	0.00017	TP2 vs TP1; TP3 vs TP1; TP4 vs TP1; TP3 vs TP2; TP4 vs TP2	1.0	0.01
	**PGE1**	0.00097	0.00232	TP3 vs TP1; TP4 vs TP1; TP3 vs TP2; TP4 vs TP2	1.0	0.01
	**Sphingosine C18:1**	0.024	0.04847	TP1 vs TP3; TP2 vs TP3	-1.0	0.01
**DPI + PMA**	**8-iso-PGE1**	0.00017	0.00180	TP3 vs TP1; TP4 vs TP1; TP3 vs TP2; TP4 vs TP2	1.0	0.01
	**8-iso-PGE2**	0.0003	0.00180	TP3 vs TP1; TP4 vs TP1; TP3 vs TP2; TP4 vs TP2	1.0	0.01
	**PGE1**	0.018	0.07216	TP3 vs TP1; TP4 vs TP1; TP3 vs TP2; TP4 vs TP2	0.60	0.40

[a] FDR: false discovery rate.
[b] Fisher's LSD: significance between the Time Points (TP) indicated (*p-value*<0.05).

Supplementary Table S3: Extracellular metabolites with significant changes during PMA and DPI+PMA treatment (n=3 samples/group) collected on Day 7.

	ANOVA				SPEARMAN'S CORRELATION	
Sample Treatment	*Compound*	*p-value*	*FDR* [a]	*Fisher's LSD* [b]	*Corr. Coefficient*	*p-value*
**PMA**	**(±)5-iPF2α-IV**	9.25E-09	1.02E-07	TP3 vs TP1; TP4 vs TP1; TP3 vs TP2; TP4 vs TP2; TP4 vs TP3	0.74	0.26
	**PGD2**	5.28E-06	2.90E-05	TP3 vs TP1; TP4 vs TP1; TP3 vs TP2; TP4 vs TP2; TP4 vs TP3	0.80	0.20
	**8-12-iPF2α-IV**	0.000158	0.00058	TP3 vs TP1; TP4 vs TP1; TP3 vs TP2; TP4 vs TP2; TP4 vs TP3	0.80	0.20
	**PGE2**	0.018996	0.05223	TP4 vs TP1; TP4 vs TP2	0.80	0.20
**DPI + PMA**	**PGD2**	2.04E-07	2.24E-06	TP3 vs TP1; TP4 vs TP1; TP3 vs TP2; TP4 vs TP2; TP4 vs TP3	0.60	0.40
	**PGE2**	3.69E-06	2.03E-05	TP3 vs TP1; TP4 vs TP1; TP3 vs TP2; TP4 vs TP2; TP4 vs TP3	0.80	0.20
	**(±)5-iPF2α-IV**	3.11E-05	0.00011	TP1 vs TP2; TP3 vs TP1; TP4 vs TP1; TP3 vs TP2; TP4 vs TP2; TP4 vs TP3	0.60	0.40
	**8-12-iPF2α-IV**	0.00027	0.00074	TP1 vs TP3; TP2 vs TP3; TP4 vs TP3	0.60	0.40

[a] FDR: false discovery rate.
[b] Fisher LSD: significance between the Time Points (TP) indicated (*p-value*<0.05).
